# Metal-assisted synthesis of unsymmetrical magnolol and honokiol analogs and their biological assessment as GABA_A_ receptor ligands

**DOI:** 10.1016/j.bmcl.2014.10.091

**Published:** 2015-01-15

**Authors:** Lukas Rycek, Roshan Puthenkalam, Michael Schnürch, Margot Ernst, Marko D. Mihovilovic

**Affiliations:** a Vienna University of Technology, Institute of Applied Synthetic Chemistry, Getreidemarkt 9/163-OC, 1060 Vienna, Austria; b Medical University of Vienna, Department of Molecular Neurosciences, Spitalgasse 4, 1090 Vienna, Austria

**Keywords:** Magnolol, Honokiol, Cross-coupling, GABA_A_, Subtype selectivity

## Abstract

We present the synthesis of new derivatives of natural products magnolol (**1**) and honokiol (**2**) and their evaluation as allosteric ligands for modulation of GABA_A_ receptor activity. New derivatives were prepared via metal assisted cross-coupling reactions in two consecutive steps. Compounds were tested by means of two-electrode voltage clamp electrophysiology at the α1β2γ2 receptor subtype at low GABA concentrations. We have identified several compounds enhancing GABA induced current (*I*_GABA_) in the range similar or even higher than the lead structures. At 3 μM, compound **8g** enhanced *I*_GABA_ by factor of 443, compared to 162 and 338 of honokiol and magnolol, respectively. Furthermore, **8g** at EC_10–20_ features a much bigger window of separation between the α1β2γ2 and the α1β1γ2 subtypes compared to honokiol, and thus improved subtype selectivity.

## Graphical abstract

**Figure:**
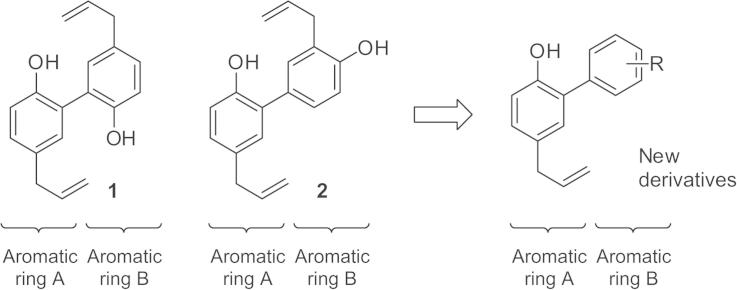

Magnolol (**1**) and honokiol (**2**) (Scheme 1) are natural products belonging to the class of neolignans isolated from *Magnolia officinalis*. They have been widely used in Eastern traditional medicine for treatment of gastric and psychiatric disorders. Therapeutic applications of Magnolia constituents were summarized comprehensively in the recent literature.^1^ Previously, several studies have revealed additional pharmaceutical effects of magnolol or honokiol as anti-angiogenetic,^2^ antiepileptic,^3^ neuroprotective,^4^ or antimicrobial and antiproliferative activity.^5^ Honokiol has been shown to have somnogenic effects in mice.^6^ Furthermore, interaction with the PPARγ receptor, involved in treatment of type 2 diabetes, metabolic syndrome, and potential anti-inflammatory target, was shown as well.^7^ In the central nervous system, neolignans interact with GABA_A_ receptors and modulate their GABA-induced activity leading to the assumption that their antiepileptic and somnogenic in vivo effects are mediated by these receptors.^8,9^

The family of GABA_A_ receptors comprises at least 26 distinct subtypes.^10^ These pentameric GABA-gated chloride channels assemble as homo- or heteropentamers in mammals from a repertoire of 19 distinct subunits that belong to eight classes. There are six α, three β, γ and ρ as well as one δ, π, ε and θ subunits. The most abundant receptor consists of two α1, two β2 and one γ2 subunits. The GABA binding site in this type of receptor is located at the extracellular interface between the beta and alpha subunits.^11^ The properties and the pharmacology of the receptor subtypes depend on the subunit composition.^12^ In addition to the site for the endogenous agonist GABA, at which other agonists or antagonists such as muscimol, bicuculline or gaboxadol also bind, there are multiple allosteric binding sites, such as the one for benzodiazepines, sites for barbiturates, neurosteroids, and several more.^11^

Magnolol and analogues have been shown to be allosteric modulators of GABA_A_ receptors, thus their binding site does not overlap with the one for GABA, muscimol, bicuculline or gaboxadol.^8,9^ The antiepileptic and somnogenic effects of magnolol and honokiol, respectively, have been connected with the benzodiazepine binding site because they can be diminished in vivo by the benzodiazepine antagonist flumazenil.^4,6^ However, neolignans and several synthetic analogs have been shown to modulate both GABA_A_ receptors with and without a benzodiazepine binding site.^8,9^ The benzodiazepine binding site requires the presence of an α and a γ2 subunit.^12^ Recently, it was shown that flumazenil also antagonizes modulation by aryl pyrazoles in receptors lacking a γ2 subunit and, thus lacking the high affinity benzodiazepine binding site.^13^ Consequently, flumazenil sensitive effects in vivo do not necessarily indicate that the benzodiazepine binding site is eliciting them.

Certain subunits have been connected with specific pharmacological effects: α1 containing receptors are thought to mediate sedative and addictive effects of benzodiazepines, while activity of ligands at receptors containing a β1 subunit have been proposed to mediate ataxic effects.^14^ With respect to GABA_A_ receptor subtypes, the natural neolignans have been demonstrated as relatively unselective compounds, and safety concerns have been raised. Subtype selective synthetic analogs have been proposed to be potentially useful.^9,13,15^

Here, we present a facile synthetic strategy towards new magnolol/honokiol derivatives and their evaluation on GABA_A_ receptors. The strength of our approach consists in a simplification of the final molecules, allowing their easy synthetic access (compared to the lead structures), while increasing the biological effect at the same time.

Magnolol and honokiol are small biaryl isomeric molecules (Scheme 1). Aromatic ring A is identical for both molecules, while ring B differs in the position of the hydroxyl group. Our approach was based on leaving the ring A untouched and carrying out simplification of the ring B. Instead of allyl and hydroxyl groups, single substituents with diverse properties were introduced in all possible positions of ring B.

Very efficiently, magnolol can be accessed by metal or enzyme mediated oxidative coupling of two 4-allylanisol units and demethylation.^16–18^ Such strategy, nevertheless, leads inevitably to the symmetric product. Unsymmetrical honokiol can be prepared by bromination of 4-allylanisole and subsequent Suzuki coupling with 4-hydroxyphenylboronic acid. Further modifications (including O-allylation, Claisen rearrangement and demethylation) lead to the desired structure.^19^ 4-Allyl-2-bromoanisole is the key intermediate which could be used within the synthesis of compounds of our interest. A drawback of this pathway is the bromination step, where overbromination of the double bond takes place and debromination with zinc is necessary, conflicting with aspects of atom efficiency. Alternatively, directed ortho-lithiation and introduction of the boronic acid into 4-allylanisole followed by subsequent coupling with aryl bromide was reported.^20,21^ However, we encountered problems with a reproduction of the protocol.

To circumvent the above outlined limitations, we have developed a simple two steps protocol, relying on palladium catalyzed transformations. In the first step, the allyl moiety is regioselectively introduced into the more reactive 4-position of 4-bromo-2-chlorophenol **3** using allylborate. In the second step, Suzuki coupling of 4-allyl-2-chlorophenol **6** with corresponding boronic acids enabled access to the products of interest. First, we focused our attention on optimization of the allylation reaction of **3** and **4** (Scheme 2). Initial experiments utilizing Pd_2_dba_3_ as a catalyst, KF as a base, dioxane/H_2_O (9:1) mixture as solvent at 150 °C (30 min) under microwave irradiation led to partial success. Three different ligands were tested (Table 1, entries 1–3) of which dppf provided most promising results. Full consumption of the starting material was not observed, however, formation of the desired product was accompanied by debromination of the starting material as side reaction, leading to 2-chlorophenol. Changing the palladium source to heterogenous PdEn40®, which can be easily separated by filtration, led to a decrease of the side reaction; however, full consumption of **3** could not be achieved (entry 4). Further investigation revealed that both conversion and side reaction can be controlled by the base used. Several bases were investigated (entries 5–9): using triethylamine or sodium acetate led to full consumption, however debromination was observed (entries 5 and 6). Slight improvement could be achieved by exploiting sodium carbonate or sodium hydroxide. However, in all cases, side product was still formed. Full conversion and complete suppression of the side reaction could be achieved finally by utilizing potassium carbonate (entry 9). Reaction time could be successfully shortened to 7 min and no sign of debromination was observed (entry 10) facilitating isolation of compound **6** in 77% of yield. Changing pinacol allylboronate **4** to potassium allytrifluoroborate **5** turned out to be beneficial for purification of the product, since pinacolester fragments often contaminated the reaction product complicating isolation. After optimization of reaction conditions target compound **6** was finally isolated in 80% (entry 11).

For the second step, we adopted a modified protocol by Denton et al.^21^ for the coupling of phenyboronic acid with chloro compound **6** under microwave irradiation; 4-allyl-2-phenylphenol **8a** was obtained in 58% of yield (Table 2, entry 1), giving an overall yield of 46%. For comparison, **8a** was also synthesized according to the above discussed pathway (demethylation/bromination of 4-allylanisole and Suzuki coupling), giving a 15% overall yield after three steps in our hands.^22^

This facile two-step protocol was subsequently employed within the preparation of a small library of compounds to obtain some preliminary structure activity relationship. Indicative substitution patterns contained weakly electron donating methyl groups in positions ortho, meta and para, strongly electron donating methoxy groups and methoxycarbonyl as well as nitro groups in all possible positions as representative electron withdrawing substituents. Only in a single case we have encountered a problem with this synthetic route: ortho-methoxycarbonyl substitution resulted in spontaneous lactonization, since a stable six-membered ring was formed (Scheme 3, **8h**). All compounds were obtained in moderate to good yields employing the outlined reaction sequence (Table 2).

The ability of the synthesized honokiol derivatives to modulate recombinantly expressed α1β2γ2 GABA_A_ receptors from rat was determined in *Xenopus laevis* oocytes (Table 2). Cells were clamped at a holding potential of −80 mV. A GABA concentration titrated to trigger 0.5% of the respective maximum GABA-elicited current of the individual oocyte (=GABA EC_0.5_) was applied together with a final concentration of 3 μM of the compound in the measurement buffer. Use of low GABA concentration allows detecting compounds exerting low efficacy modulation. Compounds were applied for 20 s. None of the compounds could open the channel without GABA, so they all are only modulators of the channel. For details on the electrophysiological recordings see Lüscher et al.^23^

Data was obtained from 5 oocytes from at least 4 different oocyte batches for the active compounds; inactives were measured with *n* = 2. The response to 3 μM honokiol (**2**) was 162 ± 31% of the response to an EC_0.5_ GABA concentration and two of the tested compounds (**8g** and **8i**) possess higher efficacy than the natural product.

A methoxy group in *meta* (**8f**) or *para* (**8g**) position is beneficial; similar behavior was encountered in case of a *m*-methoxycarbonyl group (**8i**).

Surprisingly, also high efficacy was achieved with the *o*-methyl group (**8b**). Compounds with a nitro group did not show any effect.

Since both electron donating (methyl, methoxy) as well as electron withdrawing (methoxycarbonyl) substituents can increase the efficacy, a hydrogen bond formation might be involved.

To investigate selectivity, the concentration dependent effect of **8g**, the best performing compound at 3 μM (443 ± 60% stimulation), was characterized in α1β2γ2 and α1β1γ2 receptors at a higher GABA concentration (at GABA EC_10–20_) for a better comparison with published literature (EC_10–20_),^8^ and compared with honokiol. In α1β2γ2 the EC_50_ value was at approximately 7 μM, (20 μM for honokiol) and the efficacy at 100 μM was 539 ± 98%. The EC_50_ in the β1 containing subtype is 30 μM (20 μM for honokiol), the efficacy only 206 ± 60%. The effect of **8g** in a mutant α1β2N265Sγ2 receptor was investigated at EC_0.5_ (see Supplementary Fig. 1). This mutation in the β2 subunit is known to reduce the efficacy of several compounds including loreclezole and etomidate. With **8g** we observed a pronounced drop in response, confirming the key role of β2N265 for the beta subtype selectivity. The stimulation in the wild type receptor at the same compound concentration is for comparison 2462%.

Compounds not acting on β1 receptors are thought to be non-sedative—a desired property for compounds used as anxiolytic or anticonvulsive medication (Fig. 1).^14^

In conclusion, we present a rapid and facile synthetic approach to magnolol and honokiol analogs with unsymmetrical aryl substitution employing consecutive cross coupling reactions. The protocol turned out versatile towards highly diverse aryl coupling partners.

Biological assessment of a first small product library revealed compound **8g** to possess a highly interesting combination of increased potency compared to the natural analogue in the most abundant α1β2γ2 receptor subtype, while showing strongly reduced responses in the β1 containing subtype that has been connected to ataxic effects.^14^ Such an action profile has not been reported for synthetic neolignan derivatives or the natural products, so far, underscoring the relevance of this report.^8^ It has been suggested that compounds with antiepileptic or anxiolytic properties would have fewer side effects if they are inactive in β1 containing receptor subtypes^14^ The activity difference we report here for our lead compound is much more pronounced compared to the natural products or other synthetic analogs for which this has been studied.^8,9^

Based on these preliminary findings, exploitation of the synthetic strategy towards investigation of further substitution effects in an elaborated compound library is presently underway in our laboratories.

## Figures and Tables

**Figure 1:**
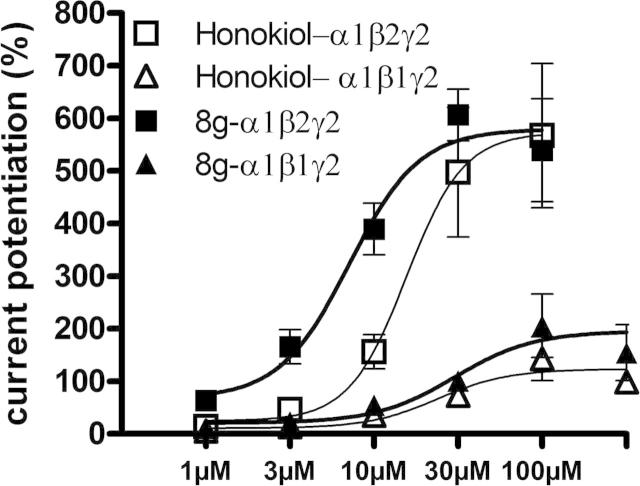
Dose–response curves for *I*_GABA_ potentiation by **8g** and honokiol in α1β1γ2 and α1β2γ2 using GABA EC_10–20_.

**Scheme 1:**
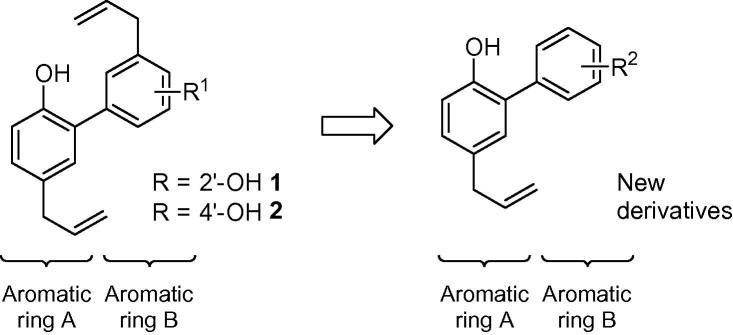
Structure of lead compounds and of new derivatives.

**Scheme 2:**
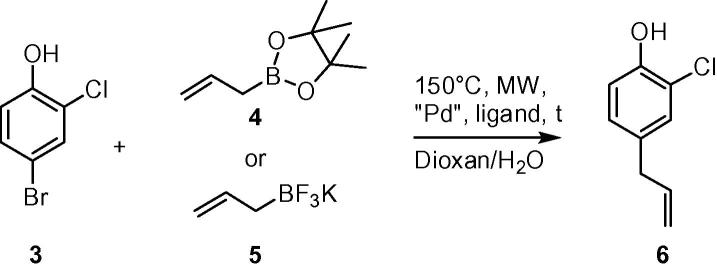
Introduction of allyl moiety.

**Scheme 3:**
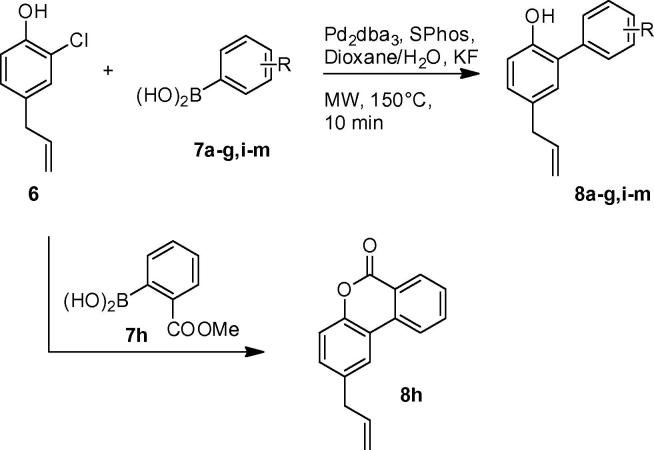
Introduction of the aryl moiety.

**Table 1:** Optimization of the reaction conditions for introduction of the allyl moiety

Entry	Pd source	Ligand	Borate	Base	Time (min)	Remaining SM	Debromination	Isolated yield (%)
1	Pd_2_dba_3_	SPhos	4	KF	30	Yes	Yes	n.d.
2	Pd_2_dba_3_	±BINAP	4	KF	30	Yes	Yes	n.d.
3	Pd_2_dba_3_	dppf	4	KF	30	Yes	Yes	n.d.
4	PdEn40	dppf	4	KF	30	Yes	Yes	n.d.
5	PdEn40	dppf	4	TEA	30	No	Yes	n.d.
6	PdEn40	dppf	4	NaOAc	30	No	Yes	n.d.
7	PdEn40	dppf	4	NaOH	30	No	Traces	n.d.
8	PdEn40	dppf	4	Na_2_CO_3_	30	No	Yes	n.d.
9	PdEn40	dppf	4	K_2_CO_3_	30	No	No	65
10	PdEn40	dppf	4	K_2_CO_3_	7	No	No	77
11	PdEn40	dppf	5	K_2_CO_3_	7	No	No	80

**Table 2:** Synthesized derivatives and their effect at α1β2γ2 GABA_A_ receptor

Compound	R	Yield	% *I*_GABA_ at 3 μM
**8a**	H	58	Inactive
**8b**	*o*-Me	34	207 ± 33
**8c**	*m*-Me	39	Inactive
**8d**	*p*-Me	46	84 ± 11
**8e**	*o*-OMe	54	Inactive
**8f**	*m*-OMe	48	136 ± 32
**8g**	*p*-OMe	34	443 ± 60
**8h**	Lactone	31	Inactive
**8i**	*m*-COOMe	34	406 ± 70
**8j**	*p*-COOMe	36	Inactive
**8k**	*o*-NO_2_	42	Inactive
**8l**	*m*-NO_2_	86	Inactive
**8m**	*p*-NO_2_	60	Inactive
**2**	—	—	162 ± 31
**1**	—	—	338 ± 93

## Supplementary data

Supplementary data Experimental procedures.
