# Corrigendum to “Metal-assisted synthesis of unsymmetrical magnolol and honokiol analogs and their biological assessment as GABA_A_ receptor ligands” [Bioorg. Med. Chem. Lett. 25/2 (2015) 400–403]

**DOI:** 10.1016/j.bmcl.2015.02.001

**Published:** 2015-04-15

**Authors:** Lukas Rycek, Roshan Puthenkalam, Michael Schnürch, Margot Ernst, Marko D. Mihovilovic

**Affiliations:** a Vienna University of Technology, Institute of Applied Synthetic Chemistry, Getreidemarkt 9/163-OC, 1060 Vienna, Austria; b Medical University of Vienna, Department of Molecular Neurosciences, Spitalgasse 4, 1090 Vienna, Austria

When this Letter was published the footnote to the first two authors was missing—it is now shown correctly here.

