# Supplementary material for: Metal-assisted synthesis of unsymmetrical magnolol and honokiol analogs and their biological assessment as GABAA receptor ligands
Source: Bioorg Med Chem Lett. 2015 Jan 15;25(2):400–3. doi: 10.1016/j.bmcl.2014.10.091 (PMC4297288; doi:10.1016/j.bmcl.2014.10.091)
Supplement: Supplementary data — Experimental procedures. [file mmc1.docx]

**Supplementary data**

Metal-Assisted Synthesis of Unsymmetrical Magnolol and Honokiol Analogs and their Biological Assessment as GABA_A_ Receptor Ligands

Lukas Rycek,^a^ Roshan Puthenkalam,^b^ Michael Schnürch,^a^ Margot Ernst,^b^
and Marko D. Mihovilovic^a*^

*^a^Vienna University of Technology, Institute of Applied Synthetic Chemistry, Getreidemarkt 9/163-OC, 1060 Vienna, Austria*

*^b^Medical University of Vienna, Spitalgasse 4, 1090 Vienna, Austria*

List of abbreviations of NMR codes and non-standard abbriviations:

s – singlet

d – doublet

t – triplet

m – multiplet

at – apparent triplet

Pd(En)30^TM^ - encapsulated palladium catalysts

dppf – 1,1'- Bis( diphenylphosphino) ferrocene

Sphos - **2-Dicyclohexylphosphino-2′,6′-dimethoxybiphenyl**

**+/-BINAP - +/-2,2′-Bis(diphenylphosphino)-1,1′-binaphthalene**

1. *Chemistry*

Microwave reactions were carried out in a BIOTAGE^®^ Initiator sixty. GC/MS spectra were measured on a Thermo Finnigan system: GC: Focus GC with a BGB5 column
(l = 30 m, d_i_ = 0.25 mm, 0.25 µm film), MS: DSQ II with quadrupol (EI). For thin layer chromatography aluminium backed silica gel 60 F254 (Merck) was used. Medium pressure liquid chromatography was performed on a Büchi Sepacore^TM^ Flash System. Pump-System: 2x Büchi Pump Module C-605, Büchi Pump Manager C-615; detector: Büchi UV Photometer C-635; fraction collector: Büchi Fraction Collector C-660. Carrier material was silica gel 60 (Merck, 40-63 µm). ^1^H- and ^13^C-NMR spectra were recorded with a Bruker AC 200 (200 MHz) or a Bruker Avance 400 (400MHz) spectrometer using CDCl_3_ as solvent. Melting points were recorded on a Büchi B-545 melting point apparatus. HRMS were measured at Shimadzu HPLC-IT-TOF mass spectrometer with either APCI or ESI ionization method.

- 1. *4-Allyl-2-chlorophenol (****6****)*

4-Bromo-2-chlorophenol (1.5 g, 7.25 mmol) was charged into the microvawe vial, together with potassium allyltriflouroborate (534.2 mg, 3.61 mmol), Pd(En)30^TM^ (300 mg, 0.05 eq.), dppf (134 mg, 0.1 eq.) and K_2_CO_3_ (667 mg, 14.5 mmol). A mixture of dioxane/water (9:1) was added (20 mL), the vial was flushed with argon and sealed. The reaction solution was heated to 150 °C for 7 minutes in a microwave oven (Biotage Initiator 60). After complete reaction the mixture was filtered through a pad of Celite, the solvent was evaporated under reduced pressure and compound **6** was purified via Kugelrohr distillation. Compound was obtained as yellowish oil in 80% (0.977 g, 5.8 mmol). ^1^H-NMR (200 MHz, CDCl_3_): δ 3.28-3.32 (d, *J* = 3.3 Hz, 2H), 5.02-5.10 (m, 2H), 5.42 (s, 1H), 5.82-6.02 (m, 1H), 7.14-7.15 6.92-7.03, (m, 2H), (d, *J =* 7.1 Hz, 1H) ^13^C-NMR (400 MHz, CDCl_3_): δ 39.2, 116.2, 116.3, 119.8, 128.7, 129.0, 133.4, 137.2 and 149.7 . B.p.: 100°C, 0.1 bar

- 1. *General procedure for Suzuki coupling*

Compound **6** (80 mg, 0.47 mmol) was charged into a microvawe vial, together with potassium fluoride (67.2 mg, 1.17 mmol), Pd_2_dba_3_ (21.5 mg, 0.05 eq.), SPhos (19.3 mg, 0.1 eq.) and the corresponding boronic acid (0.71 mmol, 1.5 eq.). A dioxane/water mixture (9:1) was added (5 mL) and the vial was flushed with argon and sealed. The reaction mixture was irradiated with microwaves at 150 °C for 10 minutes. After completion of the reaction, the reaction solution was filtered through a pad of Celite, the solvent was evaporated under reduced pressure and the crude material was absorbed onto silica gel. Purification was carried out by column chromatography using PE/EtOAc mixture (0-5% gradient if not stated different).

- - 1. *4-Allyl-2-phenylphenol (****8a****)*

Compound **8a** was prepared according to the general procedure with phenylboronic acid as coupling partner.. Column chromatography was carried out with addition of 2% Et_3_N to the eluent yielding 57.3 mg, 0.27 mmol (58%) of colourless oil. ^1^H-NMR (200 MHz, CDCl_3_): δ 3.35-3.38 (d, *J* = 6.7 Hz, 2H), 5.04-5.14 (m, 3H), 5.88-6.05 (m, 1H), 6.90-6.95 (dd, *J* = 7.4 Hz and 1.3 Hz, 1H), 7.07-7.11 (m, 2H), 7.38-7.50 (m, 5H); ^13^C-NMR (400 MHz, CDCl_3_): δ 39.5 (s), 115.8 (s), 115.9 (t), 128.0 (t), 128.1 (q), 129.2 (2C, t), 129.3 (t), 129.4 (2C, t), 130.4 (t), 132.5 (q), 137.3 (q), 137.9 (t) and 150.9 (q). HRMS [M+H]^+^ *m*/*z* calcd 211.1117, found 211.1114

- - 1. *4-Allyl-2-(2’-methylphenyl)phenol (****8b****)*

Compound **8b** was prepared according to the general procedure with 2 methyphenylboronic acid as coupling partner. Column chromatography was carried out with addition of 2%Et_3_N to the eluent yielding 34 mg, 0.16 mmol (34%) of yellowish oil. ^1^H-NMR (200 MHz, CDCl_3_): δ 2.15 (s, 3H), 3.31-3.35 (d, J = 6.7 Hz, 2H), 5.00-5.10 (m, 2H), 4.63 (s, 1H), 5.86-6.06 (m, 1H), 6.88-6.91 (m, 2H), 7.05-7.10 (dd, *J* = 7.9 Hz and 2.1 Hz, 1H), 7.20-7.31 (m, 4H); ^13^C-NMR (200 MHz, CDCl_3_): δ 19.9 (p), 39.5 (s), 115.3 (t), 115.7 (s), 126.6 (t), 127.7 (q), 128.6 (t), 129.3 (t), 130.3 (t), 130.6 (t), 130.8 (t), 132.0 (q), 136.0 (q), 137.5 (q), 138.0 (t), 150.9 (q). HRMS [M+H]^+^ *m*/*z* calcd 225.1274, found 225.1263

- - 1. *4-Allyl-2-(3’-methylphenyl)phenol (****8c****)*

Compound **8c** was prepared according to the general procedure with 3 methyphenylboronic acid as coupling partner. Column chromatography was carried out with addition of 2%Et_3_N to the eluent yielding 41 mg, 0.18 mmol (39%) of yellowish oil. ^1^H-NMR (200 MHz, CDCl_3_): δ 2.43 (s, 3H), 3.35-3.38 (d, *J* = 6.7 Hz, 2H), 5.04-5.14 (m, 3H), 5.89-6.09 (m, 1H), 6.90-6.94 (dd, *J* = 7.7 Hz and 0.7 Hz, 1H, ), 7.06-7.11 (m, 2H), 7.20-7.26 (m, 3H), 7.35-7.42 (m, 1H); ^13^C-NMR (200 MHz, CDCl_3_): δ 21.6 (p), 39.6 (s), 115.7 (s), 115.8 (t), 126.2 (t), 128.2 (q), 128.8 (t), 129.3 (t), 129.3 (t), 129.9 (t), 130.3 (t), 132.4 (q), 137.2 (q), 137.9 (t), 139.2 (q), 150.9 (q). HRMS [M+H]^+^ *m*/*z* calcd 225.1274, found 225.1264

- - 1. *4-Allyl-2-(4’-methylphenyl)phenol (****8d****)*

Compound **8d** was prepared according to the general procedure with 4 methyphenylboronic acid as coupling partner. Column chromatography was carried out with addition of 2% Et_3_N to the eluent yielding 48 mg, 0.22 mmol (46%) of yellowish oil. ^1^H-NMR (200 MHz, CDCl_3_): δ 2.43 (s, 3H), 3.35-3.39 (d, *J* = 6.7 Hz, 2H), 5.04-5.15 (m, 3H), 5.89-6.10 (m, 1H), 6.90-6.95 (dd, *J* = 7.2 Hz and 1.5 Hz, 1H), 7.07-7.11 (m, 2H), 7.26-7.40 (m, 4H); ^13^C-NMR (200 MHz, CDCl_3_): δ 21.3 (p), 39.5 (s), 115.7 (s), 115.8 (t), 128.1 (q), 129.0 (2C, t), 129.1 (t), 130.1 (2C, t), 130.4 (t), 132.4 (q), 134.3 (q), 137.8 (q), 137.9 (t), 150.9 (q). HRMS [M+H]^+^ *m*/*z* calcd 225.1274, found 225.1275

- - 1. *4-Allyl-2-(2’-methoxylphenyl)phenol (****8e****)*

Compound **8e** was prepared according to the general procedure with 2 methoxyphenylboronic acid as coupling partner. Column chromatography was carried out with addition of 2%Et_3_N to the eluent yielding 61 mg, 0.25 mmol (54%) of yellowish oil. ^1^H-NMR (200 MHz, CDCl_3_): δ 3.37-3.40 (d, J = 6.7 Hz, 2H), 3.91 (s, 3H), 5.04-5.15 (m, 2H), 5.90-6.10 (m, 1H), 6.17 (s, 1H), 6.95-6.99 (d, *J* = 8.2 Hz, 1H, ), 7.03-7.16 (m, 4H), 7.33-7.44 (m, 2H); ^13^C-NMR (200 MHz, CDCl_3_): δ 39.6 (s), 56.3 (p), 111.6 (t), 115.7 (s), 117.6 (t), 122.4 (t), 126.3 (q), 127.4 (q), 129.4 (t), 129.5 (t), 131.4 (t), 132.6 (q), 132.6 (t), 137.9 (t), 152.2 (q), 155.6 (q). HRMS [M+H]^+^ *m*/*z* calcd 241.1223, found 241.1213

- - 1. *4-Allyl-2-(3’-methoxylphenyl)phenol (****8f****)*

Compound **8f** was prepared according to the general procedure with 3 methoxyphenylboronic acid as coupling partner. Column chromatography was carried out with addition of 2%Et_3_N to the eluent yielding 54 mg, 0.23 mmol (48%) of yellowish oil. ^1^H-NMR (200 MHz, CDCl_3_): δ 3.34-3.37 (d, J = 6.7 Hz, 2H), 3.84 (s, 3H), 5.03-5.13 (m, 2H), 5.87-6.07 (m, 1H), 5.20 (s, 1H), 6.89-7.10 (m, 6H), 7.35-7.43 (at, 1H); ^13^C-NMR (200 MHz, CDCl_3_): δ 39.5 (s), 55.5 (p), 113.7 (t), 114.7 (t), 115.8 (s), 115.9 (t), 121.3 (t), 127.9 (q), 129.4 (t), 130.2 (t), 130.5 (t), 132.4 (q), 137.8 (t), 138.7 (q), 150.9 (q), 160.4 (q). HRMS [M+H]^+^ *m*/*z* calcd 241.1223, found 241.1216

- - 1. *4-Allyl-2-(4’-methoxylphenyl)phenol (****8g****)*

Compound **8g** was prepared according to the general procedure with 4 methoxyphenylboronic acid as coupling partner. Column chromatography was carried out with addition of 2%Et_3_N to the eluent yielding 38 mg, 0.16 mmol (34%) of yellowish oil. ^1^H-NMR (200 MHz, CDCl_3_): δ 3.34-3.38 (d, *J* = 6.7 Hz, 2H), 3.86 (s, 3H), 5.04-5.14 (m, 3H), 5.89-6.09 (m, 1H), 6.89-7.09 (m, 5H), 7.36-7.44 (m, 2H); ^13^C-NMR (200 MHz, CDCl_3_): δ 39.5 (s), 55.5 (p), 114.8 (2C, t), 115.7 (s), 115.8 (t), 127.8 (q), 128.9 (t), 129.5 (q), 130.4 (3C, t), 132.4 (q), 137.9 (t), 150.9 (q), 159.4 (q). HRMS [M+H]^+^ *m*/*z* calcd 241.1223, found 241.1214

- - 1. *2-Allyl-6H-benzo[c]chromen-6-one (****8h****)*

Compound **8h** was prepared according to the general procedure with 2 methoxycarbonylphenylboronic acid as coupling partner.. Column chromatography was carried out with silicagel/AgNO_3_ doped silicagel (in serial conection of columns) yielding 34 mg, 0.15 mmol (31%) of yellow solid. ^1^H-NMR (200 MHz, CDCl_3_): δ 3.50-3.51 (d, *J* = 6.6 Hz, 2H), 5.12-5.16 (m, 2H), 5.97-6.05 (m, 1H), 7.31-7.32 (d, *J* = 1.2 Hz 2H), 7.57-7.61 (at, 1H), 7.81-7.85 (at, 1H), 8.13-8.15 (d, *J* = 8.1 Hz, 1H), 7.87 (s, 1H), 8.40-8.42 (d, *J* = 7.9 Hz, 1H); ^13^C-NMR (400 MHz, CDCl_3_): δ 39.9 (s), 116.7 (s), 117.9 (t), 118.0 (q), 121.4 (q), 121.8 (t), 122.6 (t), 129.0 (t), 130.8 (t), 131.1 (t), 134.9 (t), 135.0 (q), 136.5 (q), 137.0 (t), 150.0 (q), 161.5 (q). Mp:103.6-104.8°C, HRMS [M+H]^+^ *m*/*z* calcd 237.0910, found 237.0902

- - 1. *4-Allyl-2-(3’-methoxycarbonylphenyl)phenol (****8i****)*

Compound **8i** was prepared according to the general procedure with 3-methoxycarbonylphenylboronic acid as coupling partner Column chromatography was carried out with silicagel/AgNO_3_ doped silicagel (in serial conection of columns) yielding 43 mg, 0.16 mmol (34%) of yellowish oil. ^1^H-NMR (200 MHz, CDCl_3_): δ 3.35-3.38 (d, *J* = 6.7 Hz, 2H), 3.94, (s, 3H) 4.94 (s, 1H), 5.04-5.14 (m, 2H), 5.88-6.08 (m, 1H), 6.88-6.93 (dd, *J* = 6.9 Hz and 1.9 Hz, 1H), 7.08-7.13 (m, 2H), 7.50-7.60 (m, 1H), 7.67-7.73 (m, 1H), 8.03-8.11 (m, 1H), 8.16-8.18 (m, 1H); ^13^C-NMR (200 MHz, CDCl_3_): δ 39.5 (s), 52.41 (p), 115.9 (s), 116.4 (t), 127.2 (q) 129.7 (3C, 2t, 1q) 129.9 (t), 130.3 (2C, t) 130.4 (t), 132.8 (q), 137.7 (t), 142.5 (q), 150.1 (q), 167.0 (q). HRMS [M+H]^+^ *m*/*z* calcd 269.1172, found 269.1181

- - 1. *4-Allyl-2-(4’-methoxycarbonylphenyl)phenol (****8j****)*

Compound **8j** was prepared according to the general procedure with 4-methoxycarbonylphenylboronic acid as coupling partner. Column chromatography was carried out with silicagel/AgNO_3_ doped silicagel (in serial conection of columns) yielding 45 mg, 0.17 mmol (36%) of yellowish solid. ^1^H-NMR (200 MHz, CDCl_3_): δ 3.35-3.38 (d, *J* = 6.7 Hz, 2H), 3.95 (s, 3H), 4.95 (s, 1H), 5.04-5.14 (m, 2H), 5.88-6.07 (m, 1H), 6.89-6.93 (dd, *J* = 7.4 Hz and 1.3 Hz, 1H), 7.09-7.14 (m, 2H), 7.56-7.60 (m, 2H), 8.12-8.16 (m, 2H); ^13^C-NMR (200 MHz, CDCl_3_): δ 39.3 (s), 52.2 (p), 115.8 (s), 116.2 (s), 127.1 (q), 129.1 (3C, t), 129.8 (t), 130.2 (2C, t), 130.3 (t), 132.6 (q), 137.5 (q), 142.3 (q), 150.8 (q), 166.9 (q). Mp: 66.7-68.4°C, HRMS [M+H]^+^ *m*/*z* calcd 269.1172, found 269.1159

- - 1. *4-Allyl-2-(2’-nitrophenyl)phenol (****8k****)*

Compound **8k** was prepared according to the general procedure with 2-nitrophenylboronic acid as coupling partner. Column chromatography was carried out with silicagel/AgNO_3_ doped silicagel (in serial conection of columns) yielding 50 mg, 0.20 mmol (42%) of yellow oil. ^1^H-NMR (200 MHz, CDCl_3_): δ 3.35-3.49 (d, *J* = 6.7 Hz, 2H) 4.88 (s, 1H), 5.04-5.13 (m, 2H), 5.88-6.08 (m, 1H), 6.75-6.79 (d, *J* = 8.1 Hz, 1H), 7.04-7.12 (m, 2H), 7.41-7.55 (m, 2H), 7.61-7.70 (m, 1H), 7.94-7.99 (m, 1H); ^13^C-NMR (200 MHz, CDCl_3_): δ 39.4 (s), 115.8 (s), 116.0 (t), 124.3 (t), 125.0 (q), 128.5 (t), 130.0 (2C t, q), 132.8 (2t), 132.9 (q), 133.0 (t), 137.6 (t), 149.7 (q), 150.8 (q). HRMS [M-H]^-^ *m*/*z* calcd 254.0823, found 254.0813

- - 1. *4-Allyl-2-(3’-nitrophenyl)phenol (****8l****)^1^*

Compound **8l** was prepared according to the general procedure with 3-nitrophenylboronic acid as coupling partner. Column chromatography was carried out with silicagel/AgNO_3_ doped silicagel (in serial conection of columns) yielding 103 mg, 0.40 mmol (86%) of yellowish solid. ^1^H-NMR (200 MHz, CDCl_3_): δ 3.36-3.40 (d, *J* = 6.7 Hz, 2H), 5.02 (m, 3H), 5.88-6.08 (m, 1H), 6.85-6.90 (dd, *J* = 6.4 Hz and 2.5 Hz, 1H), 7.09-7.13 (m, 2H), 7.56-7.64 (t, *J* = 7.9 Hz, 1H), 7.86-7.90 (dt, *J* = 7.8 Hz and 1.3 Hz, 1H), 8.17-8.23 (m, 1H), 8.41-8.43 (at, 1H); ^13^C-NMR (200 MHz, CDCl_3_): δ 39.4 (s), 116.1 (s), 116.5 (t), 122.2 (t), 124.4 (t), 126.0 (q), 129.5 (t), 130.2 (t), 130.7 (t), 133.2 (q), 135.5 (t), 137.5 (t), 139.7 (q), 148.5 (q), 150.8 (q). Mp:52.3-53.2°C. HRMS [M-H]^-^ *m*/*z* calcd 254.0823, found 254.0817

- - 1. *4-Allyl-2-(4’-nitrophenyl)phenol (****8m****)*

Compound **8m** was prepared according to the general procedure with 4-nitrophenylboronic acid as coupling partner. Column chromatography was carried out with silicagel/AgNO_3_ doped silicagel (in serial conection of columns) yielding 72 mg, 0.28 mmol (60%) of yellow oil. ^1^H-NMR (200 MHz, CDCl_3_): δ 3.36-3.40 (d, *J* = 6.7 Hz, 2H), 4.90 (s, 1H), 5.11-5.15 (m, 2H), 5.87-6.07 (m, 1H), 6.86-6.90 (dd, *J* = 6.5 Hz and 2.5, 1H), 7.12-7.15 (m, 2H), 7.69-7.74 (d, *J* = 8.8, Hz 2H), 8.28-8.32 (d, *J* = 8.8 Hz, 2H); ^13^C-NMR (400 MHz, CDCl_3_): δ 39.4 (s), 116.2 (s), 116.6 (t), 124.0 (2C, t), 126.2 (q), 130.2 (2C, t), 130.5 (t), 130.7 (t), 133.3 (q), 137.4 (t), 144.8 (q), 147.1 (q), 150.8 (q). HRMS [M-H]^-^ *m*/*z* calcd 254.0823, found 254.0828

1. *^1^H and ^13^C NMR spectra of synthesized compounds*


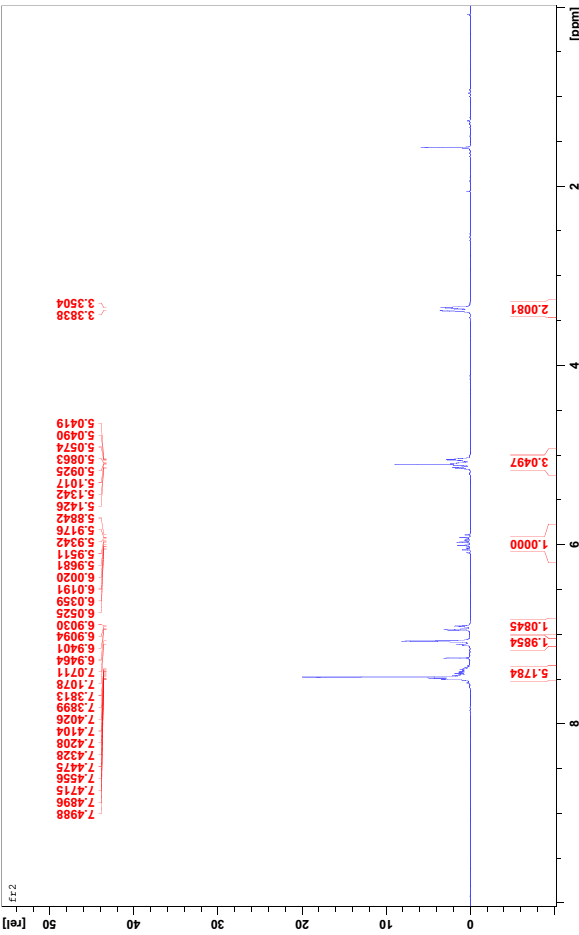

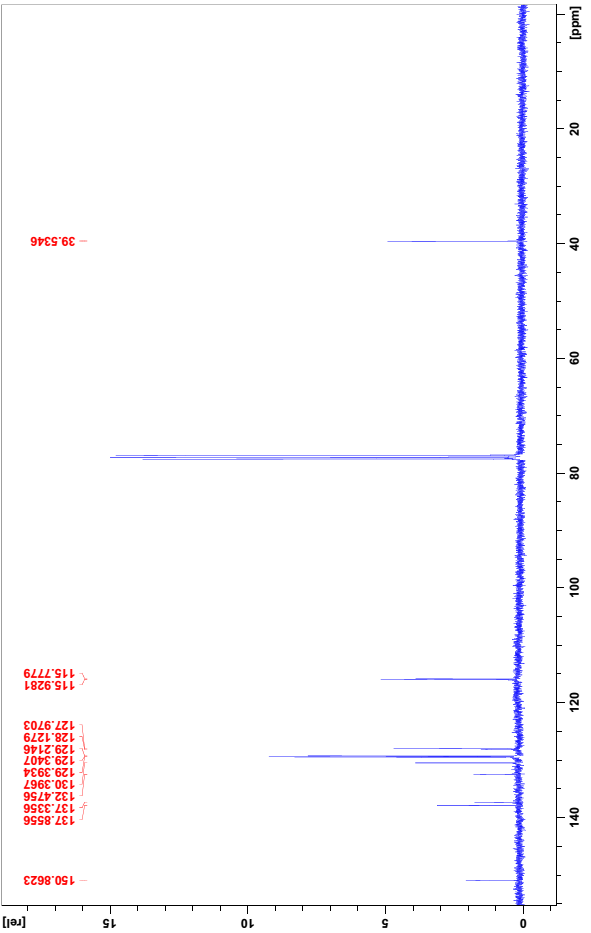

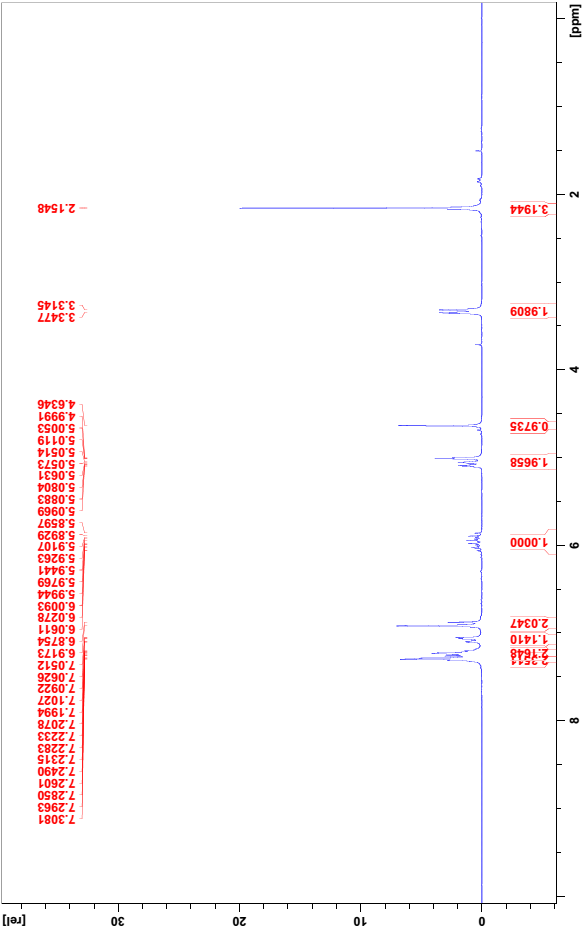

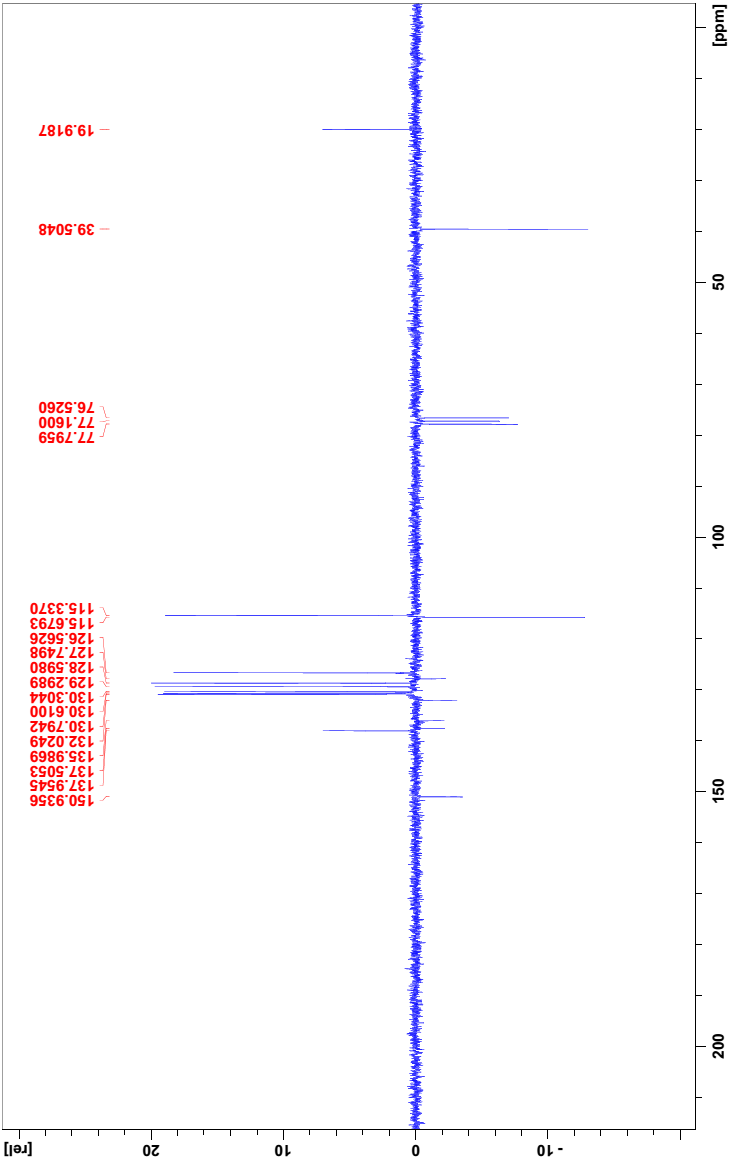

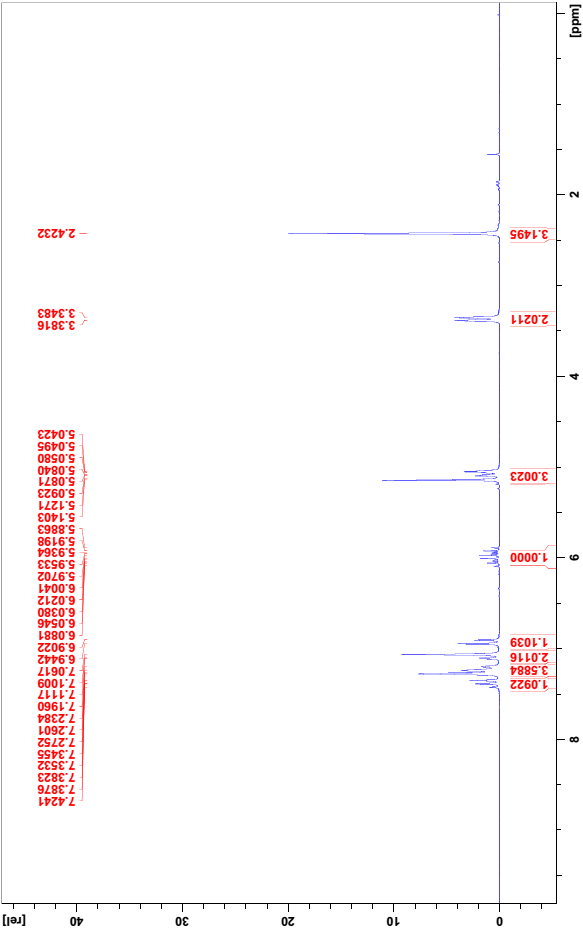

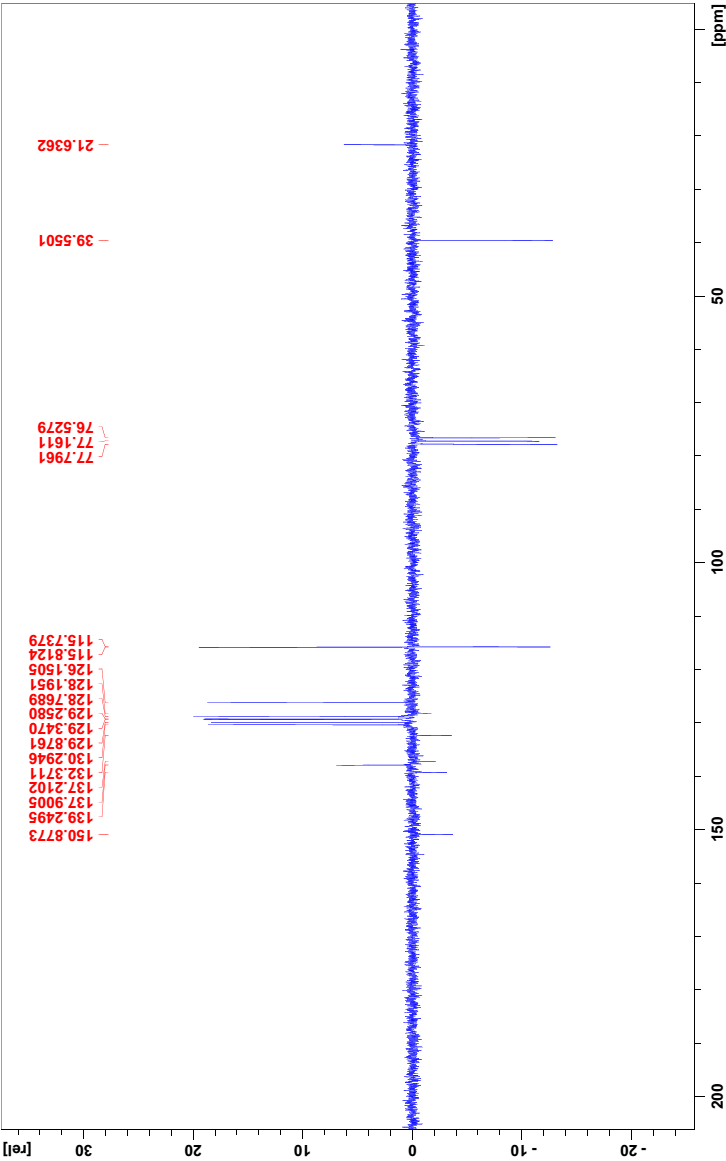

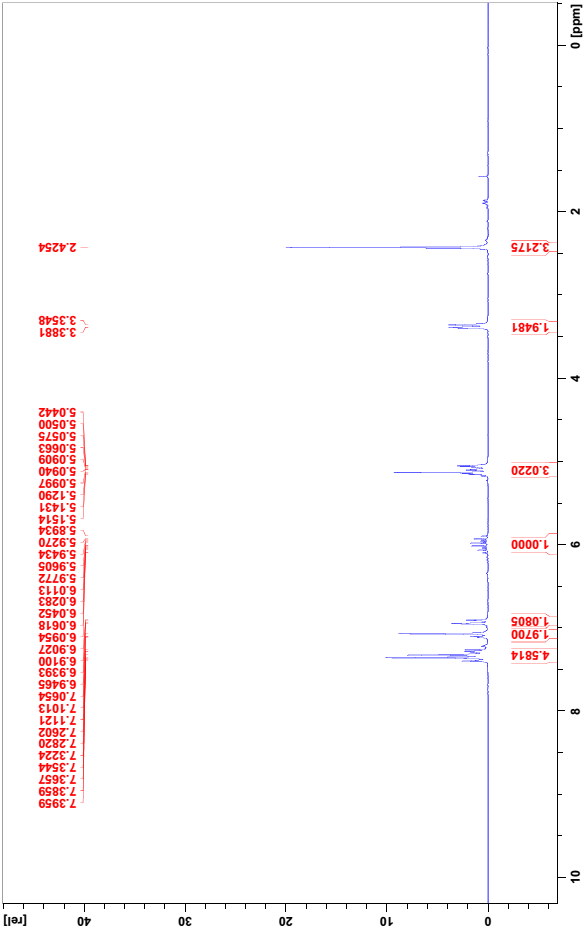

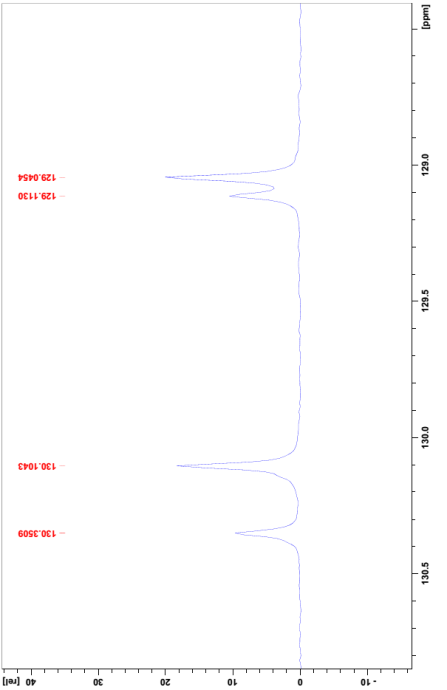

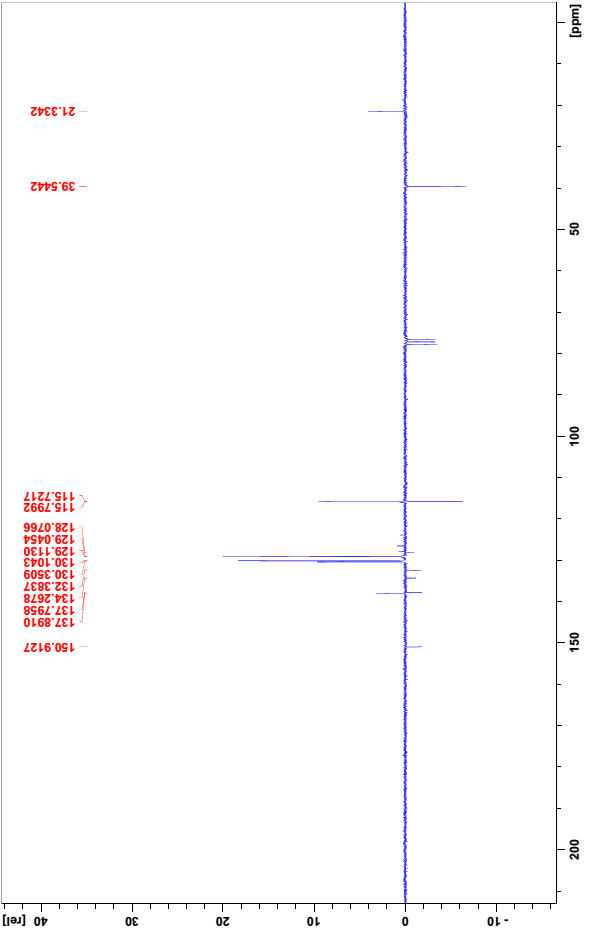

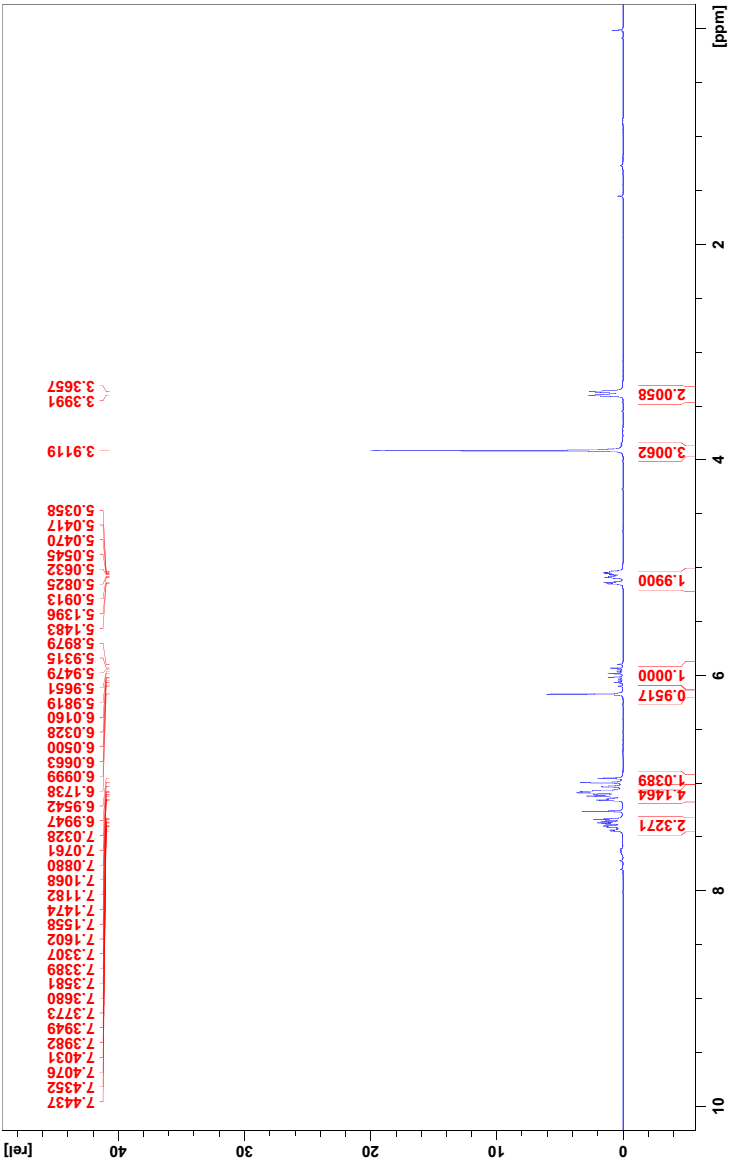

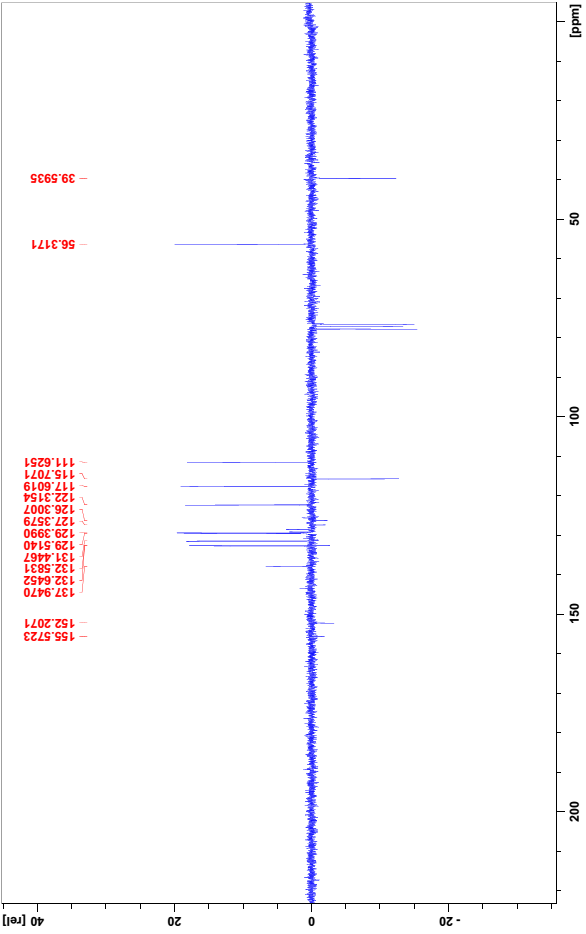

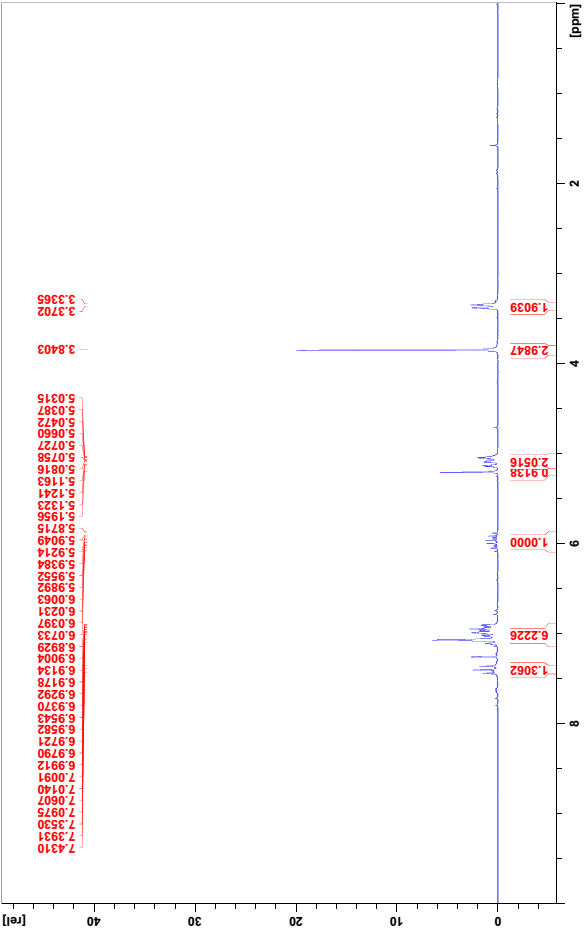

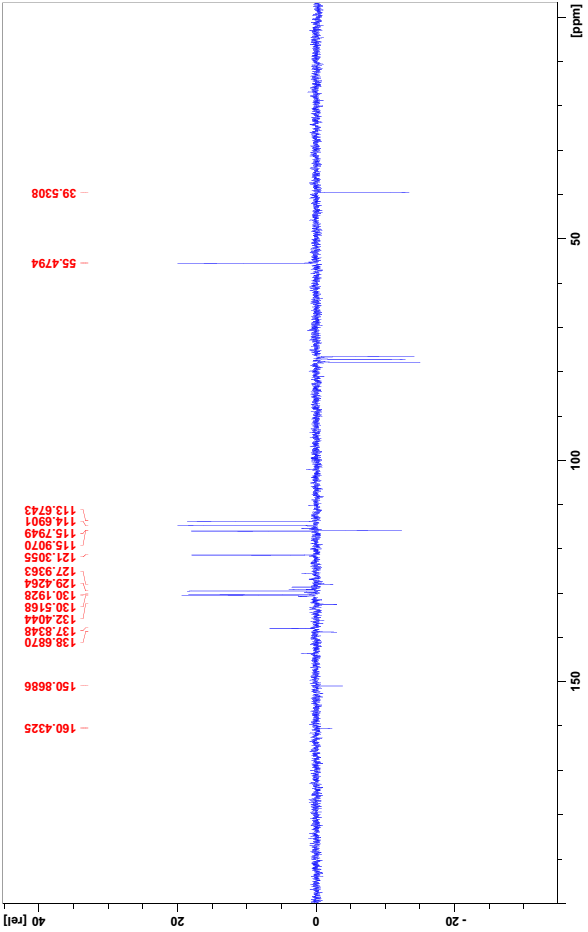

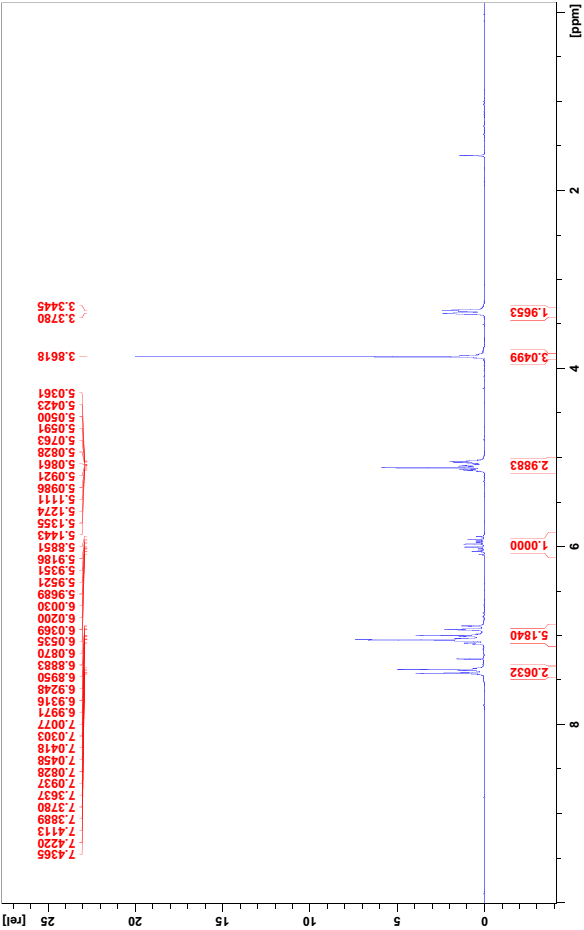

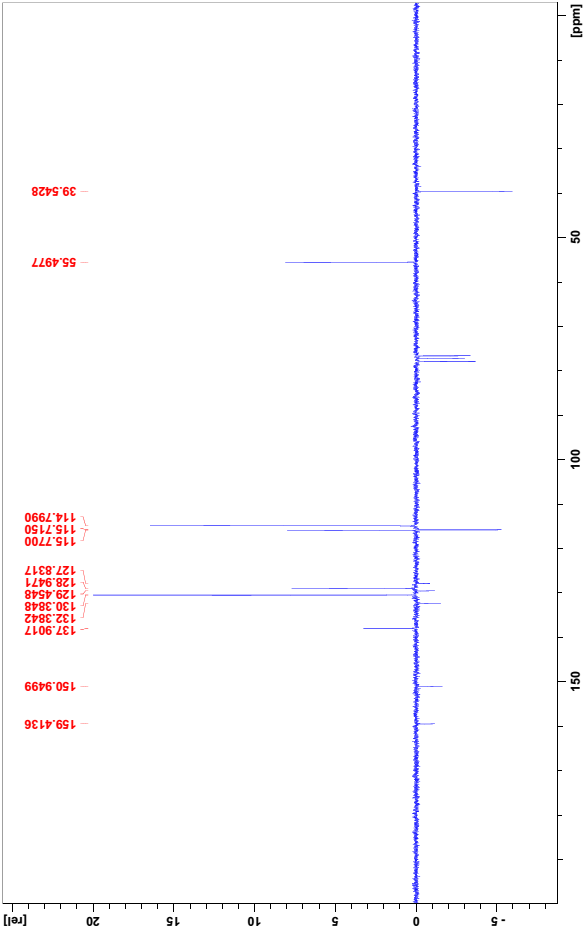

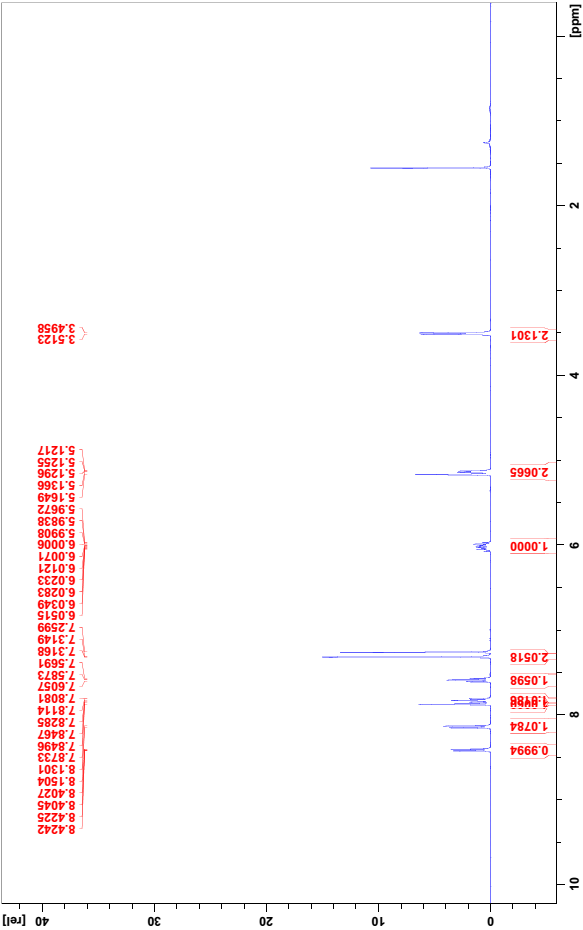

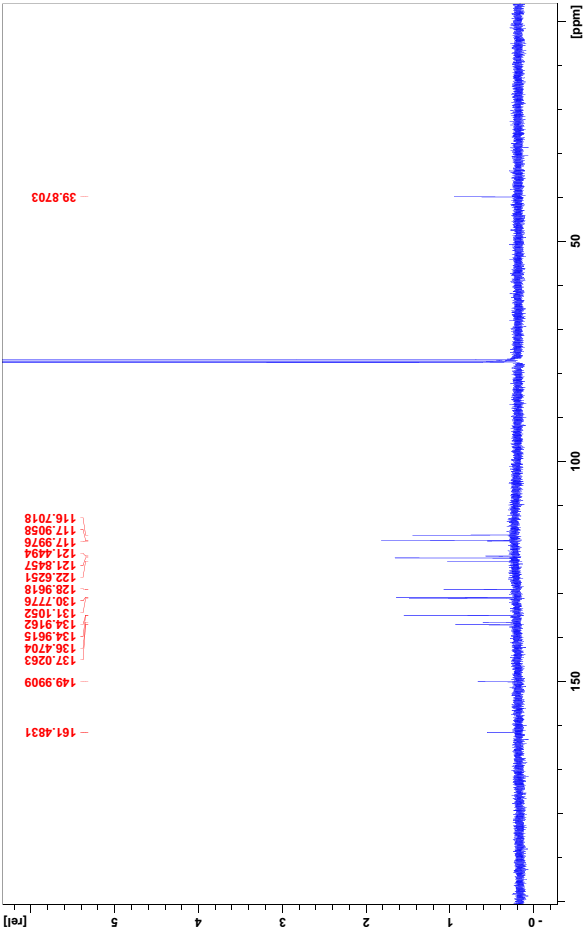

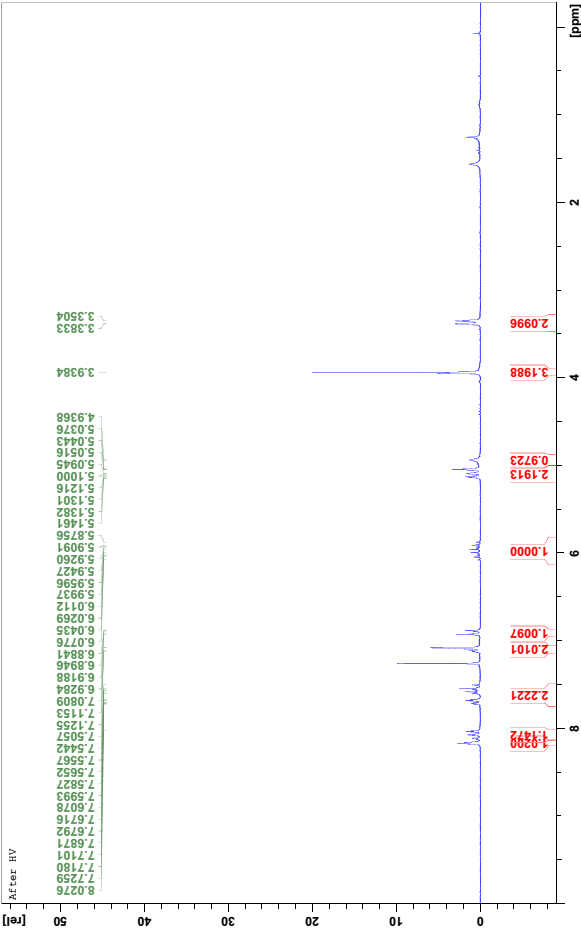

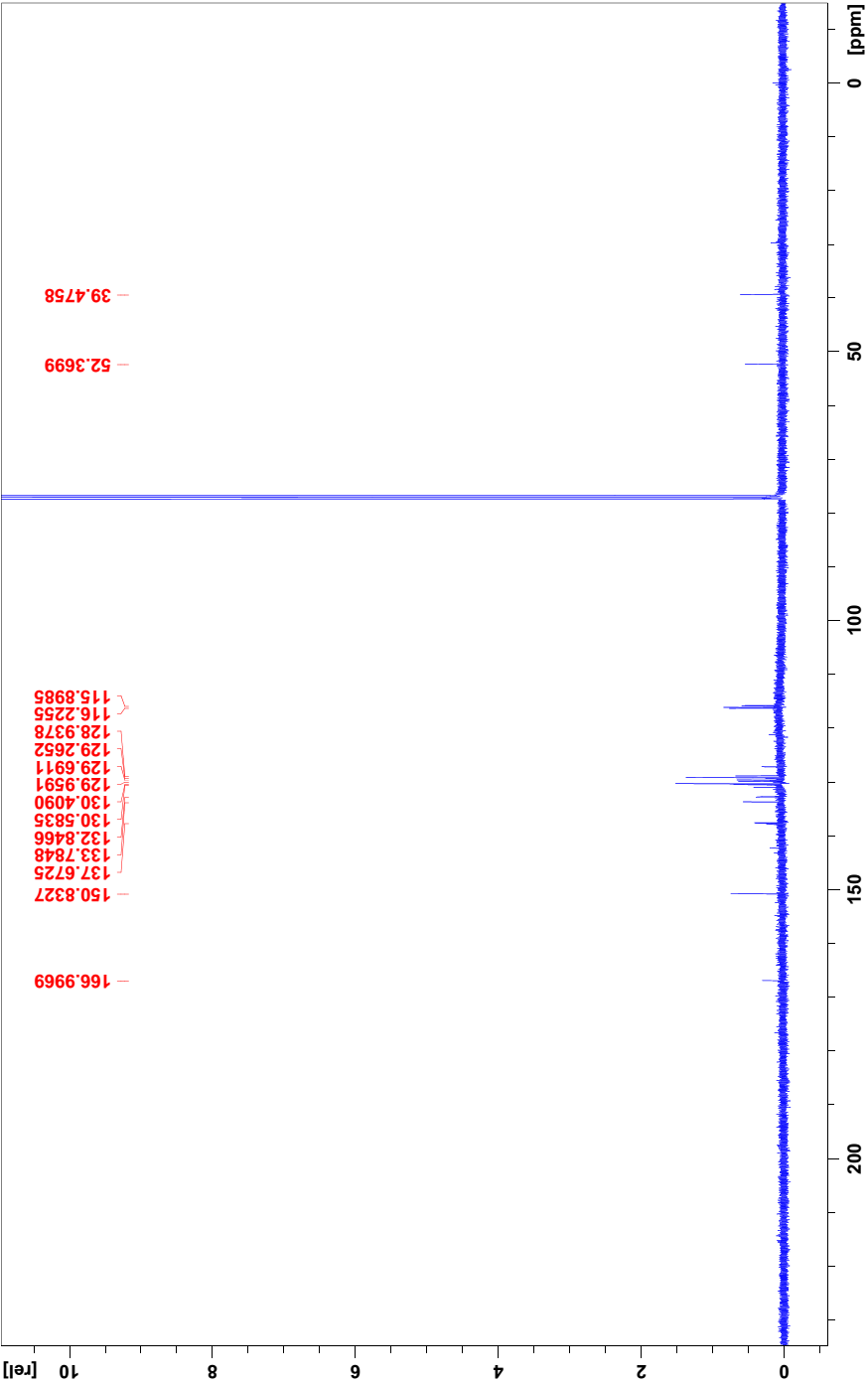

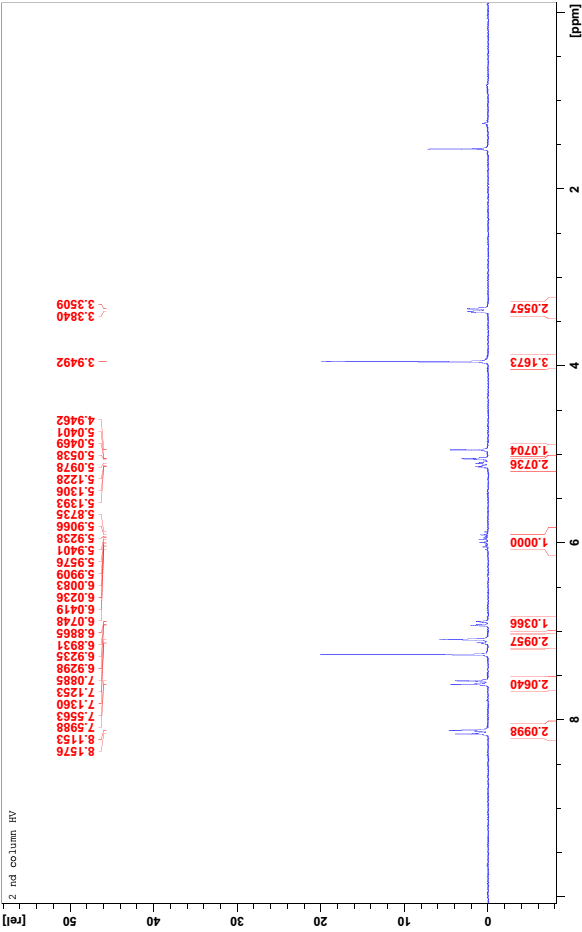

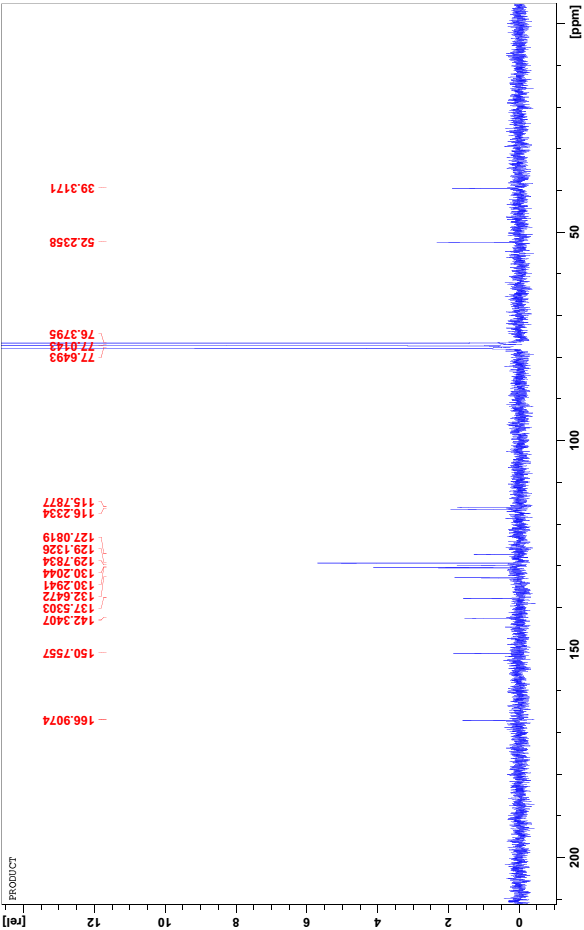

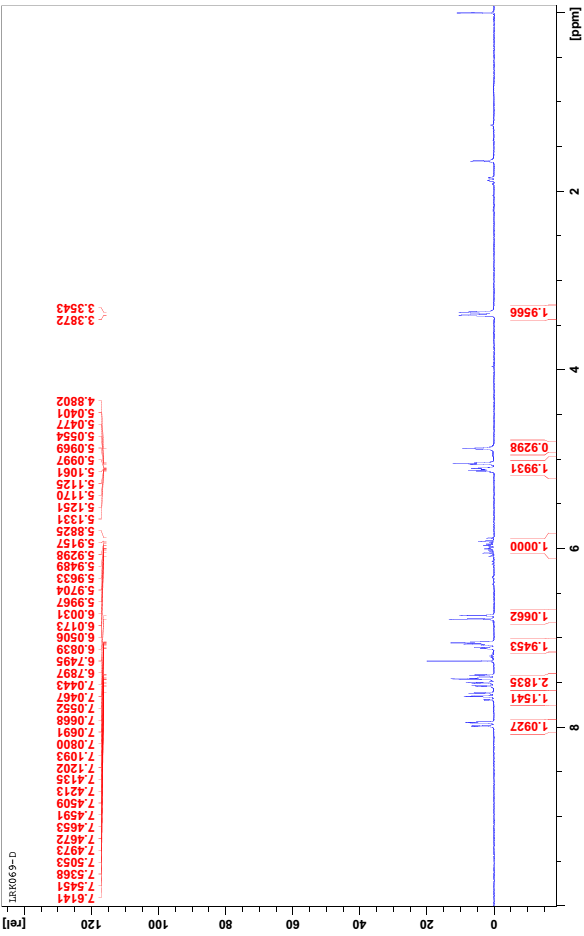

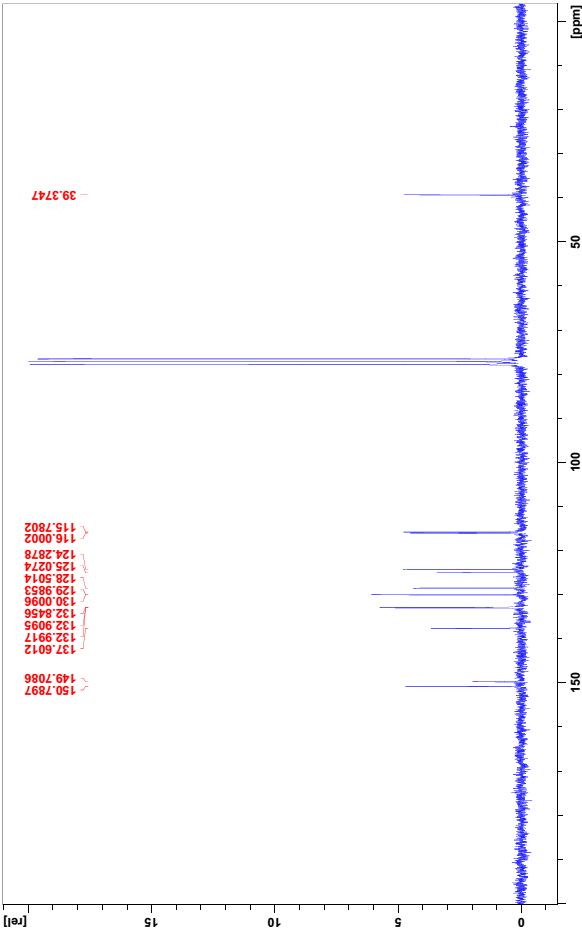

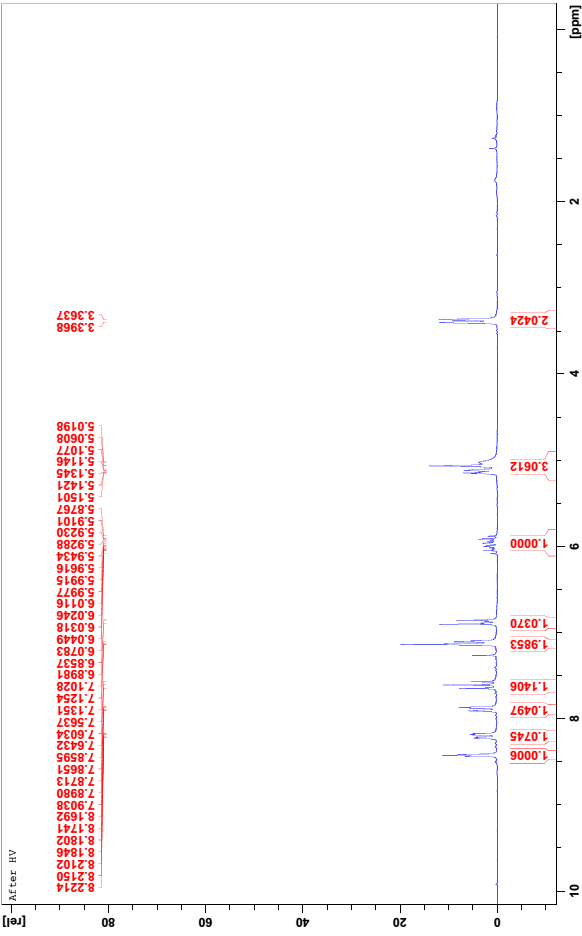

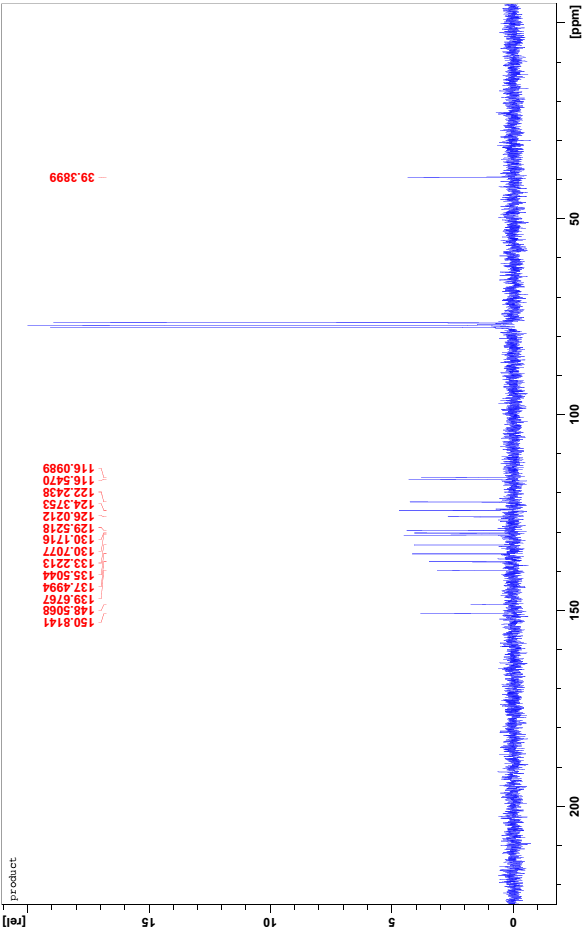

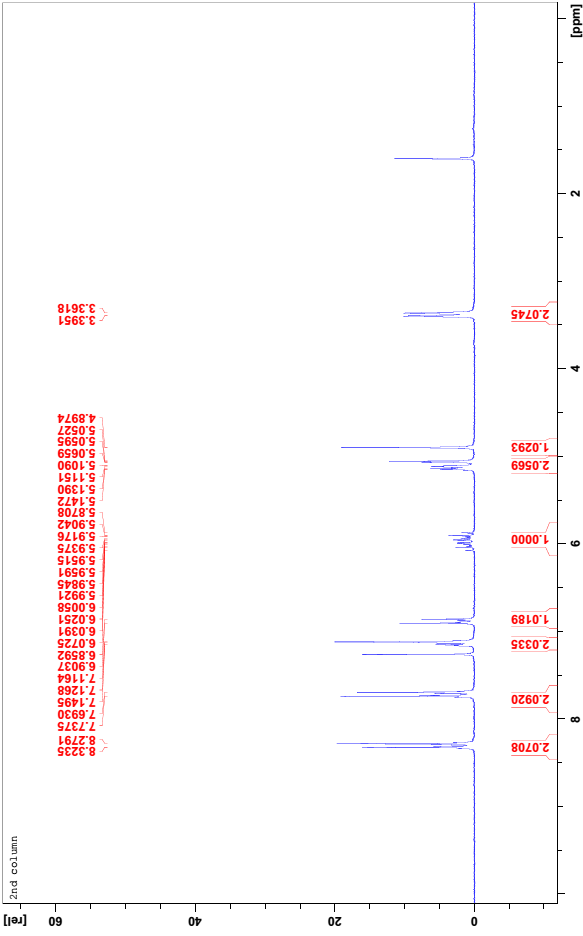

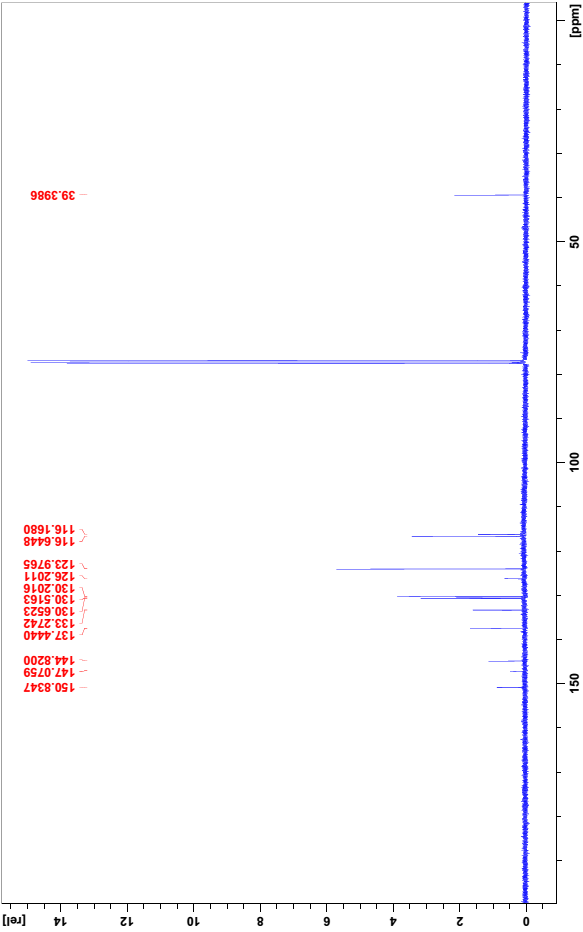

1. *Expression of recombinant GABA_A_ receptor subtypes*

Recombinant GABA_A_ receptors were expressed as described previously (see Lüscher et al and references cited therein^2^). In brief, cDNA vectors encoding for the WT GABA_A_ receptor subunits α1, β1, β2 and γ2 from rat and the mutant β2N265S were linearized. With an *in vitro* transcription kit from Ambion cRNAs were generated, which were subsequently capped and polyadenylated.

Oocytes from the *Xenopus laevis* frog which were in stage 5 to 6 of the development were mechanically isolated from the ovarian lobe with a platinum wire loop.

The cells with the follicle cell layer still around were injected with RNA mixed in a ratio 1:1:5 (α1:β:γ2), with either β1, β2 or β2N265S.

The oocytes were defolliculated with collagenase treatment and a subsequent rolling of the cells with the platinum loop on the lid of a petri dish.

The cells were incubated at +18°C.

1. *Two-electrode voltage clamp electrophysiology*

The function of GABA_A_ receptor subtypes was analyzed as described previously (see Lüscher et al^2^)

The electrophysiological measurements were performed with a modified OC-725 (Warner Instruments) amplifier.

The cells were clamped at a holding potential of -80 mV. The perfusion medium consisted of 90 mM NaCl, 1 mM KCl, 1 mM MgCl_2_, 1 mM CaCl_2_, and 5 mM Na-HEPES (pH 7.4). The application of buffer and compound respectively was done with gravity flow 6 ml/min via a glass capillary placed directly above the cell.

The compounds were applied for 20s together with a GABA concentration which can elicit 0.5 % (for the screening) and 10-20% (for the dose response curves), respectively, of maximal GABA current. Between each measurement the cells were washed for 3 min with buffer.

Modulation of GABA currents is presented as % of the current induced by the EC_0.5/10-20_ of GABA alone.

Table to Figure 1. I_GABA_ potentiation by 8g and honokiol in α1β2γ2 and α1β1γ2 using GABA EC_10-20_± SEM.

1µM 3µM 10µM 30µM 100µM

Honokiol−α1β2γ2 14±2 46±4 156±33 497±123 567±137 n=4 EC50= 20µM

Honokiol−α1β1γ2 ns 13±3 35±9 73±19 143±42 n=5 EC50=20µM

8g-α1β2γ2 63±13 166±32 389±49 606±49 539±98 n=3-4(n=2 for 300 µM) EC50=7µM

8g-α1β1γ2 ns 23±3 55±10 100±14 206±60 n=4-5 EC50=30µM

**Supplementary Figure 1.** Dose-response curves for I_GABA_ potentiation by **8g** in α1β1γ2 and α1β2γ2 using GABA EC_0.5 ,_ and 1 point pharmacology in α1β2N265Sγ2 at 10µM using GABA_0.5_

Table to Supplementary Figure 1. I_GABA_ potentiation by 8g in α1β2γ2 and α1β1γ2 using GABA EC_0.5_± SEM, and 1 point pharmacology in α1β2N265Sγ2 at 10µM using GABA EC_0.5_ ± SEM.

0.1µM 0.3µM 1µM 3µM 10µM 30µM 100µM

α1β2γ2 ns ns 145±26 364±119 2462±816 4901±1623 7475±2459 n=5

α1β1γ2 ns ns ns 20±2 86±13 258±24 1042±184 n=3

α1β2N265Sγ2 359±15 n=3

Statistical analysis was done with the one-sample t-test comparing the mean with a hypothetical value of 0 and a confidence interval of P < 0.05.

Refferences

1. Ma, L.; Chen, J.; Wang, X.; Liang, X.; Luo, Y.; Zhu, W.; Wang, T.; Peng, M.; Li, S.; Jie, S.; Peng, A.; Wei, Y.; Chen, L. *Journal of Medicinal Chemistry* **2011,** *54*.

2. Luscher, B. P.; Baur, R.; Goeldner, M.; Sigel, E. *PLoS One* **2012,** *7*.
